# HyPyP: a Hyperscanning Python Pipeline for inter-brain connectivity analysis

**DOI:** 10.1093/scan/nsaa141

**Published:** 2020-10-08

**Authors:** Anaël Ayrolles, Florence Brun, Phoebe Chen, Amir Djalovski, Yann Beauxis, Richard Delorme, Thomas Bourgeron, Suzanne Dikker, Guillaume Dumas

**Affiliations:** Department of Neuroscience, Institut Pasteur, Paris, France; Child and Adolescent Psychiatry Department, Assistance Publique - Hôpitaux de Paris, Robert Debré Hospital, Paris, France; Department of Neuroscience, Institut Pasteur, Paris, France; Department of Psychology, New York University, New York City, USA; Baruch Ivcher School of Psychology, Center for Developmental Social Neuroscience, Interdiscilinary Center Herzliya, Baruch Ivcher School of Psychology, Herzliya, Israel; Department of Psychology, Bar-Ilan University, Ramat Gan, Israel; Department of Neuroscience, Institut Pasteur, Paris, France; Department of Neuroscience, Institut Pasteur, Paris, France; Child and Adolescent Psychiatry Department, Assistance Publique - Hôpitaux de Paris, Robert Debré Hospital, Paris, France; Department of Neuroscience, Institut Pasteur, Paris, France; Department of Psychology, New York University, New York City, USA; Department of Clinical Psychology, Free University Amsterdam, Amsterdam, The Netherlands; Department of Neuroscience, Institut Pasteur, Paris, France; Center for Complex Systems and Brain Sciences, Florida Atlantic University, Center for Complex Systems and Brain Sciences, Boca Raton, FL, USA; Departement of Psychiatry, Université de Montréal, Montreal, QC, Canada; Precision Psychiatry and Social Physiology laboratory, CHU Sainte-Justine Centre de Recherche, Precision Psychiatry and Social Physiology Laboratory, Montreal, QC, Canada

**Keywords:** hyperscanning, inter-brain connectivity, non-parametric statistics, analysis pipeline, python

## Abstract

The bulk of social neuroscience takes a ‘stimulus-brain’ approach, typically comparing brain responses to different types of social stimuli, but most of the time in the absence of direct social interaction. Over the last two decades, a growing number of researchers have adopted a ‘brain-to-brain’ approach, exploring similarities between brain patterns across participants as a novel way to gain insight into the social brain. This methodological shift has facilitated the introduction of naturalistic social stimuli into the study design (e.g. movies) and, crucially, has spurred the development of new tools to directly study social interaction, both in controlled experimental settings and in more ecologically valid environments. Specifically, ‘hyperscanning’ setups, which allow the simultaneous recording of brain activity from two or more individuals during social tasks, has gained popularity in recent years. However, currently, there is no agreed-upon approach to carry out such ‘inter-brain connectivity analysis’, resulting in a scattered landscape of analysis techniques. To accommodate a growing demand to standardize analysis approaches in this fast-growing research field, we have developed Hyperscanning Python Pipeline, a comprehensive and easy open-source software package that allows (social) neuroscientists to carry-out and to interpret inter-brain connectivity analyses.

## Introduction

**Fig. 1.**
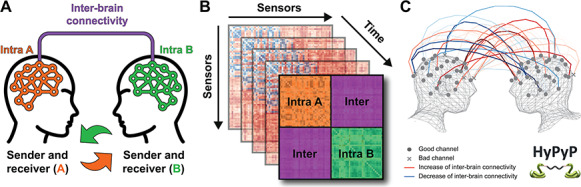
Example of a hyperscanning application of the HyPyP toolbox for the study of dyadic social interactions. (A) Schematic representation of a hyperscanning setup with two participants engaging in reciprocal social interaction. (B) Recordings are split in epochs across time, and brain signals are converted into connectivity matrices containing both intra- and inter-brain connectivity measures. (C) By comparing groups of dyads or conditions, statistical modulation of the inter-brain connectivity can be visualized (illustration generated using HyPyP. See section ‘Visualization’).

Social cognition involves the integration of biological, behavioral and social processes at both intra- and inter-individual levels (Fairhurst and Dumas, 2019). Paradoxically, most of social neuroscience research has investigated social cognition in isolated individuals exposed to pre-recorded social stimuli, in the absence of any real-time interpersonal dynamics (Hoehl and Markova, 2018). As a result, much remains unknown about how the human brain supports dynamic social interactions (Pfeiffer *et al.*, 2013; Matusz *et al.*, 2019). Neuroscientists recently developed new tools to directly study social interaction, both in controlled experimental settings and in more ecological valid environments (Hoehl and Markova, 2018; Redcay and Schilbach, 2019; Figure 1A). Specifically, ‘hyperscanning’ setups are increasingly used to simultaneously record brain activity from two or more individuals during social tasks (Czeszumski *et al.*, 2020) and to investigate the co-variations in their brain activity related to their socio-behavioral interactions (Dumas *et al.*, 2011; Babiloni and Astolfi, 2014; Czeszumski *et al.*, 2020). Hyperscanning has been used to study neural synchronization in a wide-range of social interaction contexts, ranging from parent–infant gaze communication (Leong *et al.*, 2017) to teacher–students classroom interactions (Dikker *et al.*, 2017). However, there is currently no agreed-upon approach to carry out such ‘inter-brain connectivity analysis’, resulting in a scattered landscape of analysis techniques (Burgess, 2013). To accommodate these growing demands of standardized analysis approaches in a fast-growing research field, we developed Hyperscanning Python Pipeline (HyPyP), a comprehensive and easy to understand open-source software package that allows (social) neuroscientists to carry-out and interpret a wide range of inter-brain connectivity analyses. HyPyP can handle data from groups of two or more participants, with data collected either in a simultaneous hyperscanning recording context (Dumas *et al.*, 2011) or in a non-simultaneous setup (Dmochowski *et al.*, 2014). HyPyP encourages the community to share their analyses script in open source and to provide supporting documentation for each analysis measure to motivate its use to probe specific psychologically relevant processes. As such, we hope to serve as a platform where consensus can be reached within the research community with respect to which analysis approach is best suited for which research context.

## From a stimulus-brain approach to a brain-brain approach

Over the last two decades, social and cognitive neuroscience research has increasingly moved towards more naturalistic paradigms, using a variety of recording techniques including functional magnetic resonance imaging (fMRI), electroencephalography (EEG), magneto- encephalography and functional near-infrared spectroscopy (fNIRS). This was initially triggered by two needs: capturing the social brain in the context of daily life and capturing how being actively engaged in interaction is different from passively processing social stimuli (Hari and Kujala, 2009). Various, parallel, attempts were made to tackle the challenge to study social interaction directly, emphasizing multiple sub-dimensions. Some researchers, for instance, focused on the embodied and enactive aspects of social cognition (Thompson and Varela, 2001). Interactional dynamics are then grounded in the bi-directional sensorimotor coupling of people (De Jaegher *et al.*, 2010); the dyad thus becomes a two-body dynamical system (Dumas *et al.*, 2011). Others emphasized instead how our social cognition is fundamentally different in the interactive context, even if the interaction is not reciprocal. In other words, taking a second person perspective (Schilbach *et al.*, 2013) or acting in a we-mode (Gallotti and Frith, 2013) changes our social brain. Interestingly, this delineates a two-dimensional space with one axis denoting offline *vs* online social cognition (Redcay and Schilbach, 2019), recognizing that one can mentalize and engage in metacognition about others without necessarily interacting in real-time with them (Sebanz *et al.*, 2006; Gallotti and Frith, 2013; Shea *et al.*, 2014), and another axis denoting the synchronized (i.e. symmetric) *vs* complementary (i.e. asymmetric) roles of people engaged in the interaction (Dumas *et al.*, 2010; Konvalinka *et al.*, 2014).

Beyond this ‘interactive turn’, social neuroscience has also followed a call for more naturalistic studies and ecological validity, to bring daily life into the lab, and even the lab into daily life (Dikker *et al.*, 2019). Various researchers in the past few years have highlighted the need for such ‘real-world’ neuroscience studies for a range of reasons, including (a) the need to test the laboratory model of human social cognition in dynamic naturalistic contexts, (b) to address questions that might not be straightforwardly answered in a laboratory environment and (c) to reach populations that might not otherwise be easily studied (Matusz *et al.*, 2019; Shamay-Tsoory and Mendelsohn, 2019). This move toward conducting neuroscience research in ‘real-world’ social settings has been made possible in part by the development of wireless EEG devices (Debener *et al.*, 2012). Portable, affordable technology has enabled researchers to record brain activity from people ‘in the wild’, ranging from professional environments (Toppi *et al.*, 2016), to artistic contexts (Cruz-Garza *et al.*, 2017), and even inside classrooms (Dikker *et al.*, 2017; Bevilacqua *et al.*, 2019). This move is also supported by translational psychiatry where the social context and the environment can strongly affect how patients behave (Dumas *et al.*, 2014; Bilek *et al.*, 2017), especially for neurodevelopmental disorders (Leong and Schilbach, 2019; Markova *et al.*, 2019).

Given these developments, it is no surprise that the hyperscanning technique (Montague *et al.*, 2002) has gained considerable popularity, revealing new challenges for the study of interacting social brains (Konvalinka and Roepstorff, 2012; Babiloni and Astolfi, 2014). The associated multi-brain neuroscience mixed real-time hyperscanning studies—with focus on social interaction—and post-recording multi-brain analyses—with focus on naturalistic perception, e.g. neurocinema. First, fMRI results demonstrated how the brain dynamics are indeed different when humans are taking others into account in an interactive context (King-Casas *et al.*, 2005) and how social dimension of natural stimuli tends to enhance similarity between brains (Hasson *et al.*, 2012). Hyperscanning studies using either EEG or fNIRS studies then demonstrated how specific neuromarkers are associated with ongoing social coordination (Tognoli *et al.*, 2007) and how a reciprocal interaction with others could bring the similarity of brain patterns to synchronization at the sub-second level (Dumas *et al.*, 2010). These observations have since then been extended to various social tasks, even without any rhythmic coordination (Goldstein *et al.*, 2018), thus demonstrating that inter-brain connectivity may be more than a signature of sensorimotor entrainment (Fairhurst and Dumas, 2019). Inter-brain connectivity may also constitute a marker of shared understanding (Schippers *et al.*, 2010) or cooperation (Astolfi *et al.*, 2020), and studies moving from dyads to groups also support how it is a robust signature of shared attention (Dmochowski *et al.*, 2014; Dikker *et al.*, 2017). Inter-brain connectivity is thus broader than hyperscanning, and the HyPyP library aims at supporting its use not only for simultaneous recording (Hasson *et al.*, 2012; Poulsen *et al.*, 2017) but also across a large range of measures. The HyPyP toolbox is illustrated in Figure 1. HyPyP tools can be used to perform multiple analyses to explore neural synchronizations at both intra- and inter-individual levels. These are discussed later in the overview of functionalities.

## Pipeline description

### Software specifications

The HyPyP library provides a suite of Python tools to manipulate hyperscanning data and inter-brain connectivity measures. Using a community-driven perspective, the code is open source, licensed under a three-clause Berkeley Software Distribution (BDS) license and editable at this address: https://github.com/GHFC/HyPyP.

 Running HyPyP requires Python 3.7 (or higher) with major data science libraries, such as Scipy (Virtanen *et al.*, 2020), scikit-learn (Pedregosa *et al.*, 2011), Pandas (McKinney, 2010) and Matplotlib (Hunter, 2007). HyPyP also takes advantage of other community-driven libraries, such as the MNE library (Gramfort *et al.*, 2013) for the handling of M/EEG signals and Autoreject (Jas *et al.*, 2016) for the pre-processing and rejection of artifacts. Lastly, some metrics supporting the connectivity measures also rely on the Astropy package (Collaboration *et al.*, 2013; Price-Whelan *et al.*, 2018).

### Overview of the functionalities

**Fig. 2.**
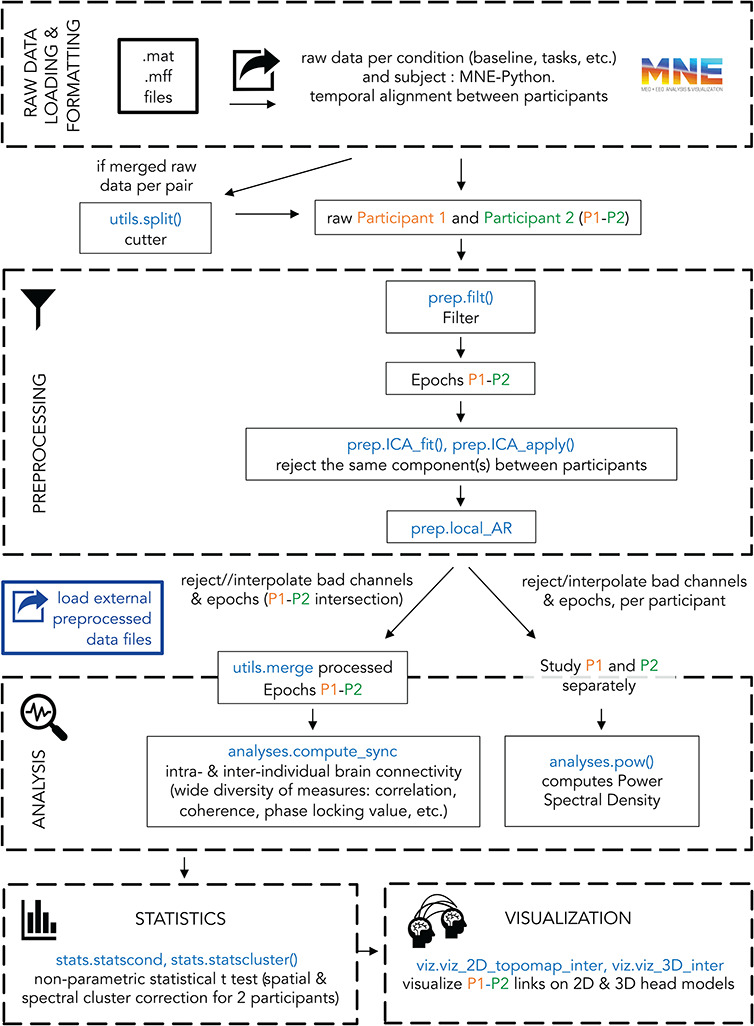
Analysis pipeline for EEG datasets in HyPyP. Steps shown in blue are module-specific, extending functionalities offered by the Python-MNE environment, in coherence with the workflow.

The HyPyP toolbox is designed to be integrated with MNE-Python (Gramfort *et al.*, 2013), a software package that enables comprehensive M/EEG data analysis at the intra-brain level. HyPyP implements these analyses at an inter-brain level (Figure 1). You can find the complete documentation with an application programming interface description on ‘HyPyP Docs’ at the link (https://hypyp.readthedocs.io). A detailed tutorial with a toy-dataset is also available on the Github page (http://github.com/GHFC/HyPyP) and illustrates what the current version of HyPyP allows researchers in social neuroscience to do. It especially covers the following analysis steps for multi-person datasets (Figure 2):

#### Load raw data.

Raw data files for each condition and participant need to be converted into epochs with the appropriate MNE function before using HyPyP. Epochs are one of the most common ways to analyze EEG signals; the MNE ecosystem has a dedicated object containing signals in a data array and all the associated parameters information—such as channel names, bad channels, frequency, sample frequency—in a metadata dictionary. Below, we illustrate how to load epochs from an example EEG dataset of two participants with the mne.read_epochs() function.

# Loading data files

epochs1 = mne.read_epochs(os.path.join('..','data', "participant1-epo.fif"), preload=True)

epochs2 = mne.read_epochs(os.path.join('..','data', "participant2-epo.fif"), preload=True)

#### Pre-process data.

HyPyP includes tools to automatically pre-process data, but researchers are still strongly encouraged to manually inspect data and determine the most appropriate pre-processing strategy. Users can also import already pre-processed data (from EEGLAB for exemple) and proceed directly to the analysis steps with HyPyP. [prep.ICA_fit, prep.ICA_choice_comp, prep.AR_local] is an adaption of MNE-Python (Gramfort *et al.*, 2013) and Autoreject (Jas *et al.*, 2016) functions, taking epochs and returning them cleaned. This process involves rejecting bad epochs, rejecting or interpolating partially bad channels per participant, and then removing the same channels and the same epochs across participants. Thus, only channels and epochs that are ‘good’ for all the participants are preserved. Independent component analysis (ICA) removal is also matched between participants such that similar independent components (ICs) are rejected across participants. To improve decomposition quality (Winkler *et al.*, 2015), the function filt removes slow drifts from raw data, then after epoching, ICA_fit prepares the signals by computing a global rejection threshold with Autoreject for bad channel rejection and pruning of highly artifacted epochs, and fits an ICA on the remaining set of epochs. ICA_choice_comp then plots ICs for each participant, lets the user choose the relevant component used as model for artifact rejection and applies ICA on epoch. AR_local applies local Autoreject in the second step:

# high-pass filtering

filt_raws = prep.filt(raws=[raw1, raw2]

# computing global AR and ICA on epochs

icas = prep.ICA_fit(epochs=[epochs1, epochs2],n_components=15, method='infomax', fit_params=dict(extended=True),random_state=42)

# selecting components semi-automatically and remove them

cleaned_epochs_ICA = prep.ICA_choice_comp(icas, epochs=[epochs1, epochs2])

This is followed by a prompt asking which participant should be used as a template and which IC from this participant should be used as a template.

# Applying local AR for each participant rejecting bad epochs, rejecting or interpolating partially bad channels per participant, and removing the same bad channels and epochs across participants.

cleaned_epochs_AR = prep.AR_local(cleaned_epochs_ICA, verbose=True)

#### Merge/split data.

[utils.merge] takes epochs from each participant to align and merge them into a single data file (whether participant data were recorded in one file or in separate files). This is particularly important when users have loaded their data into MNE and just want to concatenate multiple participants in the same MNE structure. For creating a hyper-dataset combining two recordings stored in epochs called epochs1 and epochs2, this is as simple as:

hyper_epo = merge(epochs_S1=epochs1, epochs_S2=epochs2)

[utils.merge] also takes previously pre-processed recordings: users can load data directly to visualize them (bad channels are still taken into account).

Respectively, [utils.split] takes a single hyper-epoch with both participants’ data merged and channel names indicating the participants 1 & 2 with “_1” and “_2”, and split it into two single-participant epochs:

epochs1, epochs2 = split(hyper_epo)

#### Data analysis.

[analyses.pow] computes Welch power spectral density (PSD) from pre-processed epochs. fmin and fmax set the minimum (fmin) and maximum (fmax) frequencies over which PSD is computed. The parameter n_fft states the length of the fast Fourier transform (FFT), and n_per_seg states the length of each Welch segment. The exact frequency bins are calculated based on FFT parameters and are returned in freq_list. Here is a guide for determining n_fft and n_per_seg: when n_fft is None, n_per_seg determines the sample count of each segment for computing the PSD. A longer segment means higher frequency resolution and lower time resolution. N_fft is used only if a zero-padded FFT is desired, and it has to be bigger or equal to n_per_seg. The segment length for FFT is thus n_per_seg if n_fft is None or n_fft if it is not. To estimate the power in the frequencies of interest, the segment length should be set to at least four times the period of the minimum frequency (e.g. if fmin is 4 Hz and the sampling rate is 1000 Hz, n_per_seg should be at least 1000, i.e. 1 second long). The user can either average PSD values over epochs (epochs_average = True) or preserve the complete time course. In the example below, the frequency-band-of-interest is restricted to Alpha_Low, frequencies for which PSD is actually computed are returned in freq_list and PSD values are averaged across epochs:

psd1 = analyses.pow(preproc_S1, fmin=7.5, fmax=11,

n_fft=1000, n_per_seg=1000, epochs_average=True)

psd2 = analyses.pow(preproc_S2, fmin=7.5, fmax=11,

n_fft=1000, n_per_seg=1000, epochs_average=True)

data_psd = np.array([psd1.psd, psd2.psd])

[analyses.compute_freq_bands] and [analyses.compute_sync] take pre-processed epochs, the analytic signal, and return different measures of inter-individual brain connectivity (see Roadmap Table 2 for the metrics that are implemented). The resulting matrix from the connectivity analysis is a matrix of size (2 × channel count, 2 × channel count) and represents connectivities between every pair of channels among the two participants. Indexing the matrix yields four blocks of size (channel count, channel count). Thus, two of the four blocks represent inter-brain connectivities from participant A to B and from B to A, and the other two represent intra-brain connectivity within each individual, respectively (See Figure 1B). Most of the connectivity metrics implemented are not directional, and therefore the two inter-brain connectivity ‘blocks’ are exactly symmetrical. However, this is not true for causality measures (partial directed coherence [PDC] and transfer entropy), where connectivity from A to B and from B to A is different. Similarly to PSD, the user can either average the connectivity values over epochs (epochs_average = True) or preserve the complete time course.

**Table 1.** Inter-brain connectivity measures. References related to inter-brain level are in bold. Stationarity is defined as the stability of core characteristics of the time series (e.g. mean, variance, spectral characteristics) relative to the timescale of the analyses

			Linear/Non-linear	Requires stationarity
Category	Connectivity analysis	References	interaction	of EEG
Coherency-based	coh (coherence)	Guevara and Corsi-Cabrera, 1996; **Dikker*et al.***, **2017**	Linear	Yes
	imaginary_coh (imaginary coherence)	Nolte *et al.*, 2004; **Dikker*et al.***, **2019**	Linear	Yes
	wavelet_coh (wavelet coherence)	Catarino *et al.*, 2013; **Cui*et al.***, **2012**	Linear	Yes
Amplitude/envelope correlation	envelope_corr (envelope correlation)	Mehrkanoon *et al.*, 2014; Clerico *et al.*, 2015; Zamm *et al.*, 2018	Linear	Yes
	pow_corr (power correlation)	Shaw, 1984; Guevara and Corsi-Cabrera, 1996	Linear	Yes
	proj_pow_corr (projected power correlation)	Hipp *et al.*, 2012; **Dikker*****et al.***, **2019**	Linear	Yes
Phase synchrony	ccorr (circular correlation)	**Burgess, 2013**; **Goldstein*et al.***, **2018**	Non-linear	Yes
Causality measures	plv (phase-locking value)	Lachaux *et al.*, 1999; **Dumas*et al.***, **2010**	Non-linear	No
	pli (phase lag index)	Stam *et al.*, 2007; Stam *et al.*, 2007	Non-linear	No
	pdc (partial directed coherence; granger causality)	Baccalá and Sameshima, 2001; Fallani *et al.*, 2010	Non-linear	Yes
	transfer_entropy (transfer entropy)	Schreiber, 2000; Kraskov *et al.*, 2004	Non-linear	No
Other analyses	cross-frequency analysis	Clerico *et al.*, 2015; Müller and Lindenberger, 2014	-	-
	lagged connectivity	Stephens *et al.*, 2010	-	-

**Table 2.** HyPyP roadmap of the core features

Category	Pipeline functionality	HyPyP alpha	HyPyP beta	HyPyP release
Data type	EEG/MEG	**✔**	**✔**	✔
	Fnirs	-	-	✔
Pre-processing	Load raw datasets	✔	✔	✔
	Pre-process data	✔	✔	✔
	Load previously pre-processed data	✔	✔	✔
	Temporally align datasets	✔	✔	✔
Data analysis (see also Roadmap Table 3)	Compute inter-brain connectivity	✔	✔	✔
	Compute intra-brain connectivity	✔	✔	✔
	Compute power spectral density	✔	✔	✔
	Sources reconstruction	-	-	✔
Statistical analysis (See also Roadmap Table 4)	Group-mean differences	✔	✔	✔
	Integrate behavioral variables	-	✔	✔
	Integrate intra-brain analyses	-	✔	✔
	Integrate group analysis	-	-	✔
Visualization	Visualize inter-brain connectivity	✔	✔	✔
	Visualize intra-brain analysis	-	✔	✔
	Visualize group connectivity	-	-	✔

First, the analytic signal per frequency band is computed, after which frequency- and time-frequency-domain connectivity is calculated. In the example below, circular correlation coefficient (‘ccorr’) is used. The results are then sliced to generate the inter-brain part of the matrix. This is exemplified below using Alpha_Low (frequencies); Cohens’ D is computed for further analyses.

complex_signal = analyses.compute_freq_bands

(data=[preproc_S1, preproc_S2], freq_bands)

result = analyses.compute_sync(complex_signal,

mode='ccorr')

n_ch = len(epochs1.info['ch_names'])

theta, alpha_low, alpha_high, beta, gamma = result[:, 0:n_ch,

n_ch:2*n_ch]

values = alpha_low

values -= np.diag(np.diag(values))

C = (values - np.mean(values[:])) / np.std(values[:])

This process can also be applied to intra-individual brain connectivity to support single participant analysis. In addition, the mode argument in the function can take different connectivity measurements (see section ‘Inter-brain connectivity measures’). This generates connectivity matrices for each epoch (Figure 1B).

Similar to the inter-brain analyses, results are sliced to generate the intra-brain part of the matrix, exemplified here with Alpha_Low and Cohens’ D.

for i in [0, 1]:

 theta, alpha_low, alpha_high, beta, gamma = result[:, i:i+n_ch, i:i+n_ch]

 values_intra = alpha_low

 values_intra -= np.diag(np.diag(values_intra))

 C_intra = (values_intra - np.mean(values_intra[:]))/

np.std(values_intra[:])

Cross-spectral density (CSD) values can also be sampled directly for statistical analyses:

result_intra.append(C_intra)

#### Statistics.

**Fig. 3.**
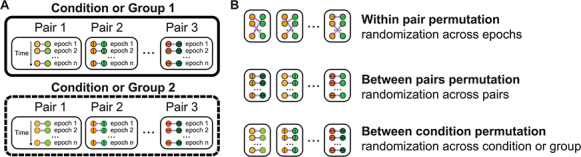
Permutation statistics for inter-brain connectivity measures. (A) Schematic representation of a hyperscanning experimental design. (B) Example of null hypothesis testing with permutation. Inter-brain connectivity measures are either calculated for the same participants but randomizing time or for fake pairs generated by randomizing pairing of participants in the same condition/group or between the condition/group.

[stats.statsCond] and [stats.statscluster] are adapted from MNE-Python statistical tests: a parametric *t*-test corrected for multiple comparisons and a non-parametric cluster-level statistical permutation test using a pre-defined threshold (alpha), corrected with channel connectivity across space and frequencies (freq_list, ch_con_freq). Both functions take PSD, intra- or inter-individual brain connectivity measurements (result or data) and return statistical values. Permutation tests can be leveraged to test a number of null hypotheses, ranging from modulation of inter-brain synchronization within dyads to between groups of participants (Figure 3). Permutation makes it possible to calculate connectivity measures either for the same participants but randomizing time or for fake pairs generated by randomizing pairing of participants in either the same condition/group or between the condition/group (Figure 3B). Clustering allows reducing familywise errors due to multiple comparisons by clustering neighboring quantities that exhibit the same effect. The neighborhood is corrected by space (adjacent sensors over the scalp) and frequencies (adjacent frequency bins). We use the test argument to define the nature of the test used to compare groups or conditions. The test can be a *t*-test for independent or paired samples (‘ind ttest’ or ‘rel ttest’), a one-way ANOVA test (‘f oneway’) or a multiple-way ANOVA test (‘f multipleway’) (if multiple-way ANOVA, the number of levels for each factor is specified with the factor_level argument).

The HyPyP simple parametric *t*-test is based on the MNE function stats.permutations_t_test() to which we added a false discovery rate correction for multiple comparisons.

statsCondTuple =

stats.statsCond(data=data_psd,epochs=preproc_S1,

n_permutations=5000, alpha=0.05)

For non-parametric cluster-based permutations, we created a matrix of a priori channel connectivity within individuals based on the channels’ position. In HyPyP, the permutation test can be used for comparing either two groups of PSD matrices or two groups of inter-brain synchrony matrices. Note that for both types of comparison, we are using the same matrix as adjacency prior, assuming both EEG were recorded with the same montage. This means that in the case of inter-brain connectivity matrix comparison, clusters of inter-brain connections are counted based on the distance between their ends on each brain according to the channel locations; in the case of PSD matrix comparison, clusters are counted based on the channel locations on one brain.

The following example is for comparing two groups’ PSD in the Alpha Low band.

con_matrixTuple = stats.con_matrix(preproc_S1,

freqs_mean=[7.5, 11])

ch_con_freq = con_matrixTuple.ch_con_freq

Below two fake groups are created for PSD comparison: one with two instances of ‘participant1’ and the other with two instances of ‘participant2’.

data_group = [np.array([psd1.psd, psd1.psd]), np.array([psd2.psd,

psd2.psd])]

statscluster=stats.statscluster(data=data_group,test=ind ttest, factor_level= None, ch_con_freq=scip.sparse.bsr_matrix(ch_con_freq), tail=0, n_permutations=5000, alpha=0.05)

The HyPyP non-parametric cluster-based permutations test can also be used to compare intra-brain connectivity values between participants. To that end, a matrix is created of a priori connectivity between channels across space and frequencies based on their position.

con_matrixTuple = stats.con_matrix(epochs=preproc_S1,

freqs_mean= np.arange[7.5, 11],

draw=False)

Note that for inter-brain connectivity measures, the resulting frequency bins are every integer frequency between fmin and fmax with a 1 Hz spectral resolution regardless of the data structure because the FFT window parameters are adaptive. For PSD, however, the resulting frequency bins are determined based on the specific FFT window parameters and are returned in ‘freq_list’. The spectral resolution may thus differ from 1 Hz depending on the parameters.

For CSD, values are averaged across each frequency, so you do not need to take frequency into account to correct clusters.

ch_con = con_matrixTuple.ch_con

Here again, two fake groups are created with twice the ‘participant1’ and twice the ‘participant2’. Here we have, for example, in the Alpha_Low band:

Alpha_low = [np.array([result_intra[0], result_intra[0]]),

np.array([result_intra[1], result_intra[1]])]

statscluster_intra = stats.statscluster(data=Alpha_Low,

test=ind ttest, factor_level= None,,

ch_con_freq = scipy.sparse.bsr_matrix(ch_con), tail=0,

n_permutations=5000, alpha=0.05)

Finally, intra-brain connectivity values can be compared to a surrogate signal. For now, creating a surrogate signal has not been implemented in HyPyP, but the user can compare intra-connectivity between subjects. No a priori connectivity between channels is considered between the two participants. In the Alpha_Low band, for example (see earlier), two fake groups are again created with twice the ‘participant1’ and twice the ‘participant2’:

data = [np.array([values, values]), np.array([result_intra[0],

result_intra[0]])]

statscluster = stats.statscluster(data=data, test=ind ttest,

factor_level = None,, ch_con_freq = None, tail=0,

n_permutations=5000, alpha=0.05)

#### Visualization.

*T* values for statistical analyses can be visualized for all channels or for significant channels only.

For example:

# visualize T values for channels for HyPyP parametric t test with FDR correction

viz.plot_significant_sensors(T_obs_plot=statsCondTuple.T_obs, epochs=preproc_S1)

# visualize T values for significant channel only for HyPyP parametric t test with FDR correction

viz.plot_significant_sensors(T_obs_plot=statsCondTuple.T_obs_plot, epochs=preproc_S1)

Statistical modulation of the inter-brain connectivity can also be visualized. [viz.viz_2D_topomap_inter, viz.viz_3D_inter] take channel locations and the matrix of inter-individual brain connectivity to visualize inter-brain links projected on either 2D topographic maps or 3D head models. Links are represented by 10th order Bezier curves; shape can be modulated with the [steps] parameter. Only values over the user-defined threshold are plotted. The sequential red color map is used for positive connectivity values, the blue for negative. Line thickness increases with the strength of the connectivity. Bad channels are excluded after pre-processing and are displayed on the visualization models with a cross (as opposed to the points that are used for good channels) to clearly distinguish between channels for which there was no significant inter-brain link and channels that were excluded prior to analysis (see Figure 1C for an example of 3D-visualization generated using HyPyP).

# Visualization of inter-brain connectivity in 2D

viz.viz_2D_topomap_inter(epochs1, epochs2, C, threshold=2, steps=10, lab=True)

# Visualization of inter-brain connectivity in 3D

viz.viz_3D_inter(epochs1, epochs2, C, threshold=2, steps=10, lab=False)

### Inter-brain connectivity measures

To measure the connectivity between two signals, we can measure the similarity in their power, phase or both (Table 1). Amplitude or envelope correlation (power correlation, envelope correlation and projected power correlation [PPC]) estimates power similarity. Phase synchrony (phase locking value [PLV], phase locking index [PLI] and circular correlation [CCorr]) measures the phase similarity, while coherency-based metrics (coherence, wavelet coherence and imaginary coherence) measure the similarity of both power and phase. Thus, the choice of appropriate connectivity metric depends on the nature of what the experimenter is interested in. For example, for neural processes, the similarity between participants’ cognitive states is better reflected in the amplitude or envelope correlation because their dynamics are on a larger time scale. For ongoing cognitive processing, with a finer-grained account of neural content and timing, the phase synchrony measures might be more relevant. Following existing hyperscanning studies and functional connectivity network studies, HyPyP natively includes common measures of phase synchrony, power synchrony, coherence-based connectivity and directed measures of casualty or information transfer (Astolfi *et al.*, 2007). We will continue adding and documenting measures on the HyPyP project page (https://pypi.org/project/HyPyP/), with the ultimate goal of formulating testable linking hypotheses between connectivity metrics and psychological processes, as such promoting consensus within the hyperscanning research field with regard to analysis choices. Here, we briefly describe the core connectivity measures that are implemented in HyPyP.

Although inter-brain connectivity metrics are often grounded in functional connectivity measures used in single-brain studies, the experimental design and underlying mechanism differ significantly. Unlike intra-brain synchrony, inter-brain synchronization is not driven by physical connections between brain sources and cannot be explained by information transfer through neuronal oscillations (Dumas *et al.*, 2012). In addition, hyperscanning studies sometimes adopt a naturalistic paradigm without trigger-locking events. These distinctions result in hyperscanning studies’ mixed methodologies and complex functional interpretation of the results.

Our toolbox addresses these different needs: HyPyP provides a variety of connectivity options, as mentioned earlier, along with functionalities to explore and compare them. First, when metrics are computed in the frequency domain, we calculate them from the analytic signal instead of spectral densities, which is more suited for nonstationary brain data in naturalistic paradigms (Lowet *et al.*, 2016). According to Lowet *et al.*, phase synchronization process is inherently non-stationary because it comes with systematic frequency variations over time, but the phase representation computed from cross-spectral density assumes stationarity. Estimating phase from the analytic signal using Hilbert transform, on the other hand, generates instantaneous phase representation, and thus does not assume stationarity. Second, a connectivity matrix is calculated for inter- and intra-brain channel pairs for all epochs (Figure 1B), resulting in a space-time-frequency representation convenient for data exploration. To facilitate a deeper understanding of the metrics, we provide an example hyperscanning dataset to test hyper-connectivities metrics. With regard to statistical tests, we include a traditional *t*-test with possibility of multiple comparisons correction through cluster-based permutation test. Future implementations will include correlation with behavioral coding, meta-analyses across different metrics and analyses of variance combining groups and conditions. On top of these analyses, we offer 2D and 3D visualizations of hyper-connections between brains and we will implement an equivalent for connectivity within brains.

Correlation and coherence are traditional linear methods to estimate brain connectivity. Correlation coefficients of blood-oxygen-level dependent signal (BOLD) signals in hyperscanning fMRI studies have been found to characterize joint attention (Koike *et al.*, 2016) and increase during social context in cinema (Hasson *et al.*, 2004). Coherence, on the other hand, is more commonly used in fNIRS and EEG studies. Wavelet coherence is commonly employed in fNIRS hyperscanning studies, such as to study cooperative and competitive behaviors (Cui *et al.*, 2012; Osaka *et al.*, 2014), imitation (Holper *et al.*, 2012), verbal communication (Jiang *et al.*, 2012; Jiang *et al.*, 2015), decision-making (Tang *et al.*, 2016) and learning (Pan *et al.*, 2018). In EEG hyperscanning, coherence has been used to study classroom social dynamics (Dikker *et al.*, 2017). As an alternative, the imaginary part of the coherence captures time-lagged synchronization only, removing zero-lagged spurious synchronizations caused by volume condition, i.e. only useful at the intra-brain level (Nolte *et al.*, 2004; Dikker *et al.*, 2019).

Non-linear brain connectivity metrics are also implemented in HyPyP, including versions of phase synchrony, power correlation and causality measures. The most commonly used phase synchrony measure is PLV, which was employed to estimate synchronization in joint action (Dumas *et al.*, 2010 2011), verbal interaction (Perez Repetto *et al.*, 2017), decision-making (Tang *et al.*, 2016) and other tasks. PLI is similar to PLV but designed for events based experimental designs. It has been adopted in a series of musical coordination studies (Lindenberger *et al.*, 2009; Sänger *et al.*, 2012). CCorr measures the covariance of phase variance between two data streams and is more robust to coincidental synchrony (Burgess, 2013) compared to PLV or PLI. CCorr has seen an increasing popularity and has been successfully implemented in studies investigating touch (Goldstein *et al.*, 2018), learning (Bevilacqua *et al.*, 2019) and language (Perez Repetto *et al.*, 2017).

Correlation between PSD and envelope in EEG data has long been used in single-brain studies (Shaw, 1984; Guevara and Corsi-Cabrera, 1996) and also adopted in hyperscanning (Zamm *et al.*, 2018). PPC, a version of power correlation between orthogonalized time series to discount spurious synchronization (Hipp *et al.*, 2012), has been found to be correlated with personality traits in naturalistic interaction (Dikker *et al.*, 2019).

Recent studies have advocated for establishing causal interpretations of synchrony values (Dean and Dunsmuir, 2016). For inferring causality in a multivariate dataset, there are autoregressive modeling and information-theoretic approaches, represented by Granger causality (GC) and transfer entropy (TE), respectively. GC and TE are based on the predictive ability of one series on the other’s future; they are equivalent under the Gaussian distribution. In EEG studies, GC is often operationalized through partial directed coherence (PDC), the frequency-domain method for GC. PDC has been found to be associated with altruistic or cooperative behaviors in hyperscanning studies (Fallani *et al.*, 2010; Astolfi *et al.*, 2012; Toppi *et al.*, 2016; Ciaramidaro *et al.*, 2018). Compared to GC, TE does not require a model and is thus more sensitive to nonlinear interactions (Schreiber, 2000). Its application in EEG hyperscanning was proposed by some groups (Liu and Pelowski, 2014) but has not yet been explored much.

Here is a list of short definitions of each metric:

- Coherence: the squared magnitude of cross-spectral density between *x* and *y*, divided by the autospectral density of *x* and *y*, respectively.
- Imaginary coherence: the absolute value of the imaginary part of the coherence.
- Wavelet coherence: the cross-correlation between two signals as a function of frequency and time, detecting time-localized synchrony in nonstationary signals.
- Envelope correlation: Pearson correlation between the envelope of two signals.
- Power correlation: Pearson correlation between the power of two signals.
- PPC: Pearson correlation between the signal envelopes after the projection of one signal on the other has been removed.
- CCorr: Pearson correlation between the angle values of two signals.
- PLV: variability of the phase difference between two signals over time.
- Phase lag index: the amount of asymmetry of the relative phase distribution (Aydore *et al.*, 2013).
- PDC (GC in the frequency domain): the amount of spectral information in the future of y that can be predicted from the past of *x*.
- TE: equivalent to GC under the Gaussian distribution, can better handle non-linearity.

## Roadmap

To our knowledge, HyPyP is the first comprehensive toolbox dedicated to quantifying brain connectivity across multiple participants. Tables 2–4 list the roadmap for HyPyP iterations. These roadmaps are subject to change and are actively maintained on the HyPyP project page (pypi.org/project/HyPyP).

**Table 3.** HyPyP roadmap of the analyses and visualization tools

Analysis & visualization	HyPyP alpha	HyPyP beta	HyPyP release
Contrast between two conditions (A vs. B)	✔	✔	✔
Display statistical map	✔	✔	✔
Simulated dataset	-	✔	✔
Integrate behavioral coding	-	✔	✔
Compare intra- & inter-individual metrics	-	-	✔
Searchlight analysis connectivity × behavior	-	-	✔
Frequency correlations	-	-	✔
High-def visualization options	-	-	✔
Meta-connectivity analysis	-	-	✔
Integrate other continuous data (e.g. physiological, movement)	-	-	✔

**Table 4.** HyPyP roadmap of the documentation

Documentation	HyPyP alpha	HyPyP beta	HyPyP release
Code documentation	✔	✔	✔
Tutorial documentation	-	✔	✔
Detailed metrics documentation	-	✔	✔
Guidelines about psychological processes associated with inter-brain metrics		-	✔

We aim to integrate the study of behavioral variables from inter to intra-brain level and extend statistical and visualization functions to group analyses. Also, taking advantage of MNE source level functionality, we aim to integrate source-level hyperscanning to complement our channel-level visualization. In addition to streamlining and implementing these functionalities (listed in Tables 2–4), we will continue releasing documentation around the pipeline, including documented code, tutorial materials and guidelines for linking hypotheses between connectivity metrics and psychological processes. The toolbox is designed with consideration for the complexity and multi-dimensionality of hyperscanning studies. By incorporating the time course, behavioral data and meta-analyses, we hope the toolbox can support the discovery of functional and behavioral correlates of connectivity metrics in multi-brain studies.

## Conclusion

The HyPyP is an analysis toolbox designed to support (social) neuroscientists interested in comparing brain data across two or more participants. It already integrates the core tools to run inter-brain connectivity measures from pre-processing to visualization and will continue to be improved upon in a community-driven fashion. The specific tools provided in HyPyP will facilitate standardized inter-individual neurophysiological analyses to support scientific progress and replicability in social neuroscience research.
